# Comparison of Seroconversion in Children and Adults With Mild COVID-19

**DOI:** 10.1001/jamanetworkopen.2022.1313

**Published:** 2022-03-09

**Authors:** Zheng Quan Toh, Jeremy Anderson, Nadia Mazarakis, Melanie Neeland, Rachel A. Higgins, Karin Rautenbacher, Kate Dohle, Jill Nguyen, Isabella Overmars, Celeste Donato, Sohinee Sarkar, Vanessa Clifford, Andrew Daley, Suellen Nicholson, Francesca L. Mordant, Kanta Subbarao, David P. Burgner, Nigel Curtis, Julie E. Bines, Sarah McNab, Andrew C. Steer, Kim Mulholland, Shidan Tosif, Nigel W. Crawford, Daniel G. Pellicci, Lien Anh Ha Do, Paul V. Licciardi

**Affiliations:** 1 Division of Infection and Immunity, Murdoch Children’s Research Institute, The Royal Children’s Hospital, Melbourne, Australia; 2 Department of Paediatrics, University of Melbourne, Melbourne, Australia; 3 Laboratory Services, The Royal Children’s Hospital, Melbourne, Australia; 4 Department of General Medicine, The Royal Children’s Hospital, Melbourne, Australia; 5 Victorian Infectious Diseases Reference Laboratory, The Royal Melbourne Hospital, Peter Doherty Institute for Infection and Immunity, Melbourne, Australia; 6 Department of Microbiology and Immunology, The University of Melbourne, Peter Doherty Institute for Infection and Immunity, Melbourne, Australia; 7 WHO (World Health Organization) Collaborating Centre for Reference and Research on Influenza, Peter Doherty Institute for Infection and Immunity, Melbourne, Australia; 8 Department of Gastroenterology, The Royal Children’s Hospital, Melbourne, Australia; 9 Department of Infectious Disease Epidemiology, London School of Hygiene and Tropical Medicine, London, United Kingdom

## Abstract

**Importance:**

The immune response in children with SARS-CoV-2 infection is not well understood.

**Objective:**

To compare seroconversion in nonhospitalized children and adults with mild SARS-CoV-2 infection and identify factors that are associated with seroconversion.

**Design, Setting, and Participants:**

This household cohort study of SARS-CoV-2 infection collected weekly nasopharyngeal and throat swabs and blood samples during the acute (median, 7 days for children and 12 days for adults [IQR, 4-13] days) and convalescent (median, 41 [IQR, 31-49] days) periods after polymerase chain reaction (PCR) diagnosis for analysis. Participants were recruited at The Royal Children’s Hospital, Melbourne, Australia, from May 10 to October 28, 2020. Participants included patients who had a SARS-CoV-2–positive nasopharyngeal or oropharyngeal swab specimen using PCR analysis.

**Main Outcomes and Measures:**

SARS-CoV-2 immunoglobulin G (IgG) and cellular (T cell and B cell) responses in children and adults. Seroconversion was defined by seropositivity in all 3 (an in-house enzyme-linked immunosorbent assay [ELISA] and 2 commercial assays: a SARS-CoV-2 S1/S2 IgG assay and a SARS-CoV-2 antibody ELISA) serological assays.

**Results:**

Among 108 participants with SARS-CoV-2–positive PCR findings, 57 were children (35 boys [61.4%]; median age, 4 [IQR, 2-10] years) and 51 were adults (28 women [54.9%]; median age, 37 [IQR, 34-45] years). Using the 3 established serological assays, a lower proportion of children had seroconversion to IgG compared with adults (20 of 54 [37.0%] vs 32 of 42 [76.2%]; *P* < .001). This result was not associated with viral load, which was similar in children and adults (mean [SD] cycle threshold [Ct] value, 28.58 [6.83] vs 24.14 [8.47]; *P* = .09). In addition, age and sex were not associated with seroconversion within children (median age, 4 [IQR, 2-14] years for both seropositive and seronegative groups; seroconversion by sex, 10 of 21 girls [47.6%] vs 10 of 33 boys [30.3%]) or adults (median ages, 37 years for seropositive and 40 years for seronegative adults [IQR, 34-39 years]; seroconversion by sex, 18 of 24 women [75.0%] vs 14 of 18 men [77.8%]) (*P* > .05 for all comparisons between seronegative and seropositive groups). Symptomatic adults had 3-fold higher SARS-CoV-2 IgG levels than asymptomatic adults (median, 227.5 [IQR, 133.7-521.6] vs 75.3 [IQR, 36.9-113.6] IU/mL), whereas no differences were observed in children regardless of symptoms. Moreover, differences in cellular immune responses were observed in adults compared with children with seroconversion.

**Conclusions and Relevance:**

The findings of this cohort study suggest that among patients with mild COVID-19, children may be less likely to have seroconversion than adults despite similar viral loads. This finding has implications for future protection after SARS-CoV-2 infection in children and for interpretation of serosurveys that involve children. Further research to understand why seroconversion and development of symptoms are potentially less likely in children after SARS-CoV-2 infection and to compare vaccine responses may be of clinical and scientific importance.

## Key Points

**Question:**

What proportion of children with mild SARS-CoV-2 infection undergo seroconversion compared with adults?

**Findings:**

In this cohort study of 57 children and 51 adults, the proportion of children with seroconversion to SARS-CoV-2 was half that found in adults despite similar viral load.

**Meaning:**

These findings suggest that serology may provide a less reliable marker of prior SARS-CoV-2 infection in children and support strategies to protect children against COVID-19, including vaccination.

## Teaser

This household cohort study compares seroconversion and cellular immunity in children and adults after infection with the ancestral (Wuhan) strain of SARS-CoV-2 and investigates the factors associated with this response in Melbourne, Australia.

## Introduction

Since the start of the COVID-19 pandemic, most children with COVID-19 either have been asymptomatic or have presented with mild illness, and very few have required hospitalization.^1,2,3^ However, COVID-19 cases in children increased in 2021 and continue to increase in 2022, likely owing to the emergence of SARS-CoV-2 variants, particularly the highly transmissible Delta and Omicron variants,^4,5,6^ as well as increased contact between children attending school. Although the severity of COVID-19 generally correlates with the magnitude of host immune responses against SARS-CoV-2,^7,8^ children and adolescents with mild or asymptomatic SARS-CoV-2 infection can also mount robust and durable antibody responses.^9^

Immunity to SARS-CoV-2 induced through natural infection is likely to be mediated by a combination of humoral and cellular immunity.^10,11,12^ Some studies comparing children and adults have revealed distinct immune profiles,^13,14,15,16^ which have been associated with less severe outcomes in children compared with adults.

The immune correlates of protection against SARS-CoV-2 have not been identified, although neutralizing antibodies are increasingly recognized as the primary mediator of protection.^17,18,19^ Most adults (>90%) infected with SARS-CoV-2 mount an immunoglobulin G (IgG) response,^20,21^ which can persist for at least 12 months.^22^ Seropositive recovered adults are estimated to have as much as 89% protection from reinfection against the same strain.^23,24^ In contrast, the proportion of children infected with SARS-CoV-2 with seroconversion is unknown, particularly among those with asymptomatic or mild infection.

Characterization of the immune response after natural infection is important to better understand factors that may be related to future protection. In this study, we compared seroconversion and cellular immunity in children and adults after infection with the ancestral (Wuhan) strain of SARS-CoV-2 and investigated the factors associated with this response in a household cohort study in Melbourne, Australia.

## Methods

### Study Design

This cohort study was conducted at The Royal Children’s Hospital, Melbourne, Australia, from May 10 to October 28, 2020. Children or adults infected with SARS-CoV-2 and their household members were invited to participate in this study. Nasopharyngeal and oropharyngeal swab specimens were collected from the participants to detect SARS-CoV-2 infection, and blood samples were collected to measure humoral responses. This study followed the Strengthening the Reporting of Observational Studies in Epidemiology (STROBE) reporting guideline for cohort studies. Written informed consent and assent were obtained from adults or parents and children, respectively. The study was approved by the Human Research Ethics Committee at The Royal Children’s Hospital.

### Study Participants

Participants were nonhospitalized patients who were asymptomatic or had mild symptoms of COVID-19 (ie, coryza, headaches, nausea, fever, cough, sore throat, malaise, and/or muscle aches). Baseline swab and convalescent blood samples (median, 41 [IQR, 31-49] days) were collected from all patients. A subset of patients had 2 to 4 additional weekly nasopharyngeal and oropharyngeal swabs and acute blood samples collected (median, 7 days for children and 12 days for adults [IQR, 4-13 days] after the baseline swab), as well as a later blood sample collected at a median of 94 (IQR, 91-100) days.

### SARS-CoV-2 Diagnosis Using Polymerase Chain Reaction Analysis

Combined oropharyngeal and nasopharyngeal (or deep nasal) swabs were collected using dry flocked swabs (FLOQSwabs; Copan). Briefly, the flocked swabs were eluted in phosphate-buffered saline (PBS), and the eluent was used for nucleic acid extraction with a commercially available system (MagNA Pure 96; Roche) according to the manufacturer’s instructions. All samples were tested with the modular SARS and Wuhan CoV E-gene kit (targeting the E-gene; sensitivity of 96.5%, specificity of 98.5%^25^) (LightMix; TIB Molbiol) using 10 μL of nucleic acid extract according to the manufacturer’s instructions. Reverse transcriptase quantitative polymerase chain reaction (PCR) analysis was performed on a real-time PCR device (LightCycler 480 II; Roche). Cycle threshold (Ct) values at diagnosis for patients with positive findings for SARS-CoV-2 are provided when available.

### SARS-CoV-2 Serological Diagnosis

#### In-house Enzyme-Linked Immunosorbent Assay Method

We used a modified, 2-step enzyme-linked immunosorbent assay (ELISA) based on the previously described Mount Sinai Laboratory method to measure SARS-CoV-2–specific IgG responses.^26,27^ Briefly, 96-well high-binding plates were coated with receptor-binding domain or S1 (Sino Biological) antigen diluted in PBS at 2 μg/mL. Serum samples were first screened with receptor-binding domain antigen; potential seropositive samples were then confirmed with S1 antigen. Goat antihuman IgG (1:10 000) horseradish peroxidase–conjugated secondary antibody was used, and the plates were developed using 3.3′, 5.5′-tetramethylbenzidine substrate solution. Seropositive samples were titrated and calculated based on a World Health Organization SARS-CoV-2 pooled serum standard (National Institute of Biological Standards and Controls). Results are reported in international units per milliliter. The cutoff for seropositivity was 8.36 IU/mL based on prepandemic samples, whereas seronegative samples were given half of the seropositive cutoff value.

#### SARS-CoV-2 S1/S2 IgG Assay

The quantitative commercial assay for the detection of IgG antibodies against S1/S2 antigens of SARS-CoV-2 was performed according to the manufacturer’s instructions (DiaSorin). Data were reported as assay units (AU) per milliliter. Negative findings were less than 12.0 AU/mL, equivocal findings were 12.0 to 15.0 AU/mL, and positive findings were greater than 15.0 AU/mL.

#### SARS-CoV-2 Antibody ELISA

This qualitative commercial ELISA (Beijing Wantai Biological Pharmacy Enterprise Co, Ltd) detects total antibodies (including IgG and IgM) to the SARS-CoV-2 receptor-binding domain antigen. The assays were performed according to the manufacturer’s instructions. Data were reported as a ratio of the absorbance over the kit control cutoff; seropositivity is defined as a ratio of at least 1.0.

#### SARS-CoV-2 Microneutralization Assay

A subset of samples (n = 12) underwent testing for neutralizing antibodies using the SARS-CoV-2 microneutralization assay. Briefly, SARS-CoV-2 isolate CoV/Australia/VIC01/202027 passaged in Vero cells was stored at −80°C. Serial 2-fold dilutions of heat-inactivated plasma were incubated with 100 of 50% tissue culture infectious dose of SARS-CoV-2 for 1 hour, and residual virus infectivity was assessed in quadruplicate wells of Vero cells; viral cytopathic effect was read on day 5. The neutralizing antibody titer is calculated using the Reed/Muench method.^28^

### Flow Cytometry

For T- and B-cell populations (from the convalescent sample obtained at a median of 41 [IQR, 31-49] days), whole blood was lysed with red blood cell lysis buffer (1:10 dilution) for 10 minutes at room temperature. Whole blood was then diluted in PBS and centrifuged at 400*g* for 5 minutes. Cells were washed once more in PBS and resuspended in 50 μL of blocking solution (1% Fc block and 5% normal rat serum in PBS) for 15 minutes on ice. After blocking, cells were washed with 1 mL of flow cytometry staining buffer (FACS) (2% fetal bovine serum in PBS) and stained with 50 μL of antibody cocktail 1 or 2 for 20 minutes on ice. The flow cytometry antibodies and the supplier are provided in eTable 1 in the Supplement. After staining, cells were washed twice and resuspended in 100 μL of FACS buffer for acquisition using flow cytometry (Aurora system; Cytek Biosciences). Compensation was performed at the time of acquisition using compensation beads (BD Bioscience). Data were analyzed using FlowJo software, version 10 (Tree Star). The manual gating strategy for B- and T-cell panels are shown in eFigure 1 and eFigure 2 in the Supplement.

For innate cell populations (acute period sample, median of 7 days for children and 12 days for adults [IQR, 4-13 days] after the baseline swab), 100 μL of whole blood was lysed with 1 mL of red cell lysis buffer for 10 minutes at room temperature. Cells were washed with 1 mL of PBS and centrifuged at 350*g* for 5 minutes. After 2 more washes, cells were resuspended in PBS for viability staining using near-infrared viability dye according to manufacturer’s instructions. The viability dye reaction was stopped by the addition of FACS buffer (2% heat-inactivated fetal calf serum in 2mM EDTA), and cells were centrifuged at 350*g* for 5 minutes. Cells were then resuspended in human FC-block for 5 minutes at room temperature. The whole-blood innate cocktail (eTable 2 in the Supplement) made up at 2× concentration was added at a dilution of 1:1 with the cells and incubated for 30 minutes on ice. After staining, cells were washed with 2 mL of FACS buffer and centrifuged at 350*g* for 5 minutes. Cells were then resuspended in 2% paraformaldehyde for a 20-minute fixation on ice, washed, and resuspended in 150 μL of FACS buffer for acquisition using a cell analyzer (LSR X-20 Fortessa; BD Biosciences). eFigure 3 in the Supplement depicts the manual gating strategy for innate cell populations.

### Statistical Analysis

Only patients who had a positive PCR result for SARS-CoV-2 or who were seropositive for SARS-CoV-2 at baseline were included in the analysis. All patients underwent testing for serological analysis, whereas a subset of patients with sufficient blood samples also underwent testing for cellular responses by flow cytometry. Patient characteristics associated with antibody responses such as viral load, age, sex, and whether symptomatic or asymptomatic (where data were available) were examined.

The antibody levels and Ct values between children and adults as well as within seropositive and seronegative children or adults were compared using the Mann-Whitney *U* test. We used the Fisher exact test to compare both the proportion who were seropositive and the proportion who were symptomatic in children and adults. For correlation analysis, antibody levels were log-transformed and analyzed using Pearson correlation analysis. For flow cytometry data, the Friedman test with a Benjamini-Hochberg post hoc test was used to account for the false discovery rate for comparison within adults and children based on their PCR and serological status. All analyses were performed with GraphPad Prism, version 7.0 (GraphPad). Two-sided *P* < .05 was considered statistically significant.

## Results

### Patient Characteristics

From May 10 to October 28, 2020, 134 children (18 years or younger) and 160 adults (aged 19-73 years) from 95 families were recruited into the household cohort study. A total of 57 children (42.5%; 22 girls [38.6%] and 35 boys [61.4%]) and 51 adults (31.9%; 28 women [54.9%] and 23 men [45.1%]) were infected with SARS-CoV-2 (defined as having a positive PCR result for SARS-CoV-2 at any of the 5 time points) and were included in our analysis; 30 of 57 children and 19 of 51 adults had 2 to 4 additional weekly swab specimens collected. Four adults had negative PCR findings at baseline but returned a positive PCR result 1 week later. The patient characteristics are described in the Table. The median ages at enrollment for children was 4 (IQR, 2-10) years; for adults, 37 (IQR, 34-45) years. No race or ethnicity data were collected.

**Table.** Patient Characteristics

Characteristic	Patients^a^	
	Children (n = 57)	Adults (n = 51)
Age at enrollment, median (IQR), y	4 (2-10)	37 (34-45)
Sex		
Female	22 (38.6)	28 (54.9)
Male	35 (61.4)	23 (45.1)
SARS-CoV-2 infection status		
RT-qPCR positive	57 (100)	47 (92.2)
RT-qPCR negative but seropositive	0	4 (7.8)
% Asymptomatic	11 (19.3)	6 (11.8)

Abbreviation: RT-qPRC, reverse transcriptase–quantitative polymerase chain reaction.

^a^ Eligible patients were seropositive based on immunoglobulin G findings. Unless otherwise indicated, data are expressed as number (%) of patients.

### Seroconversion Findings

**Figure 1.**
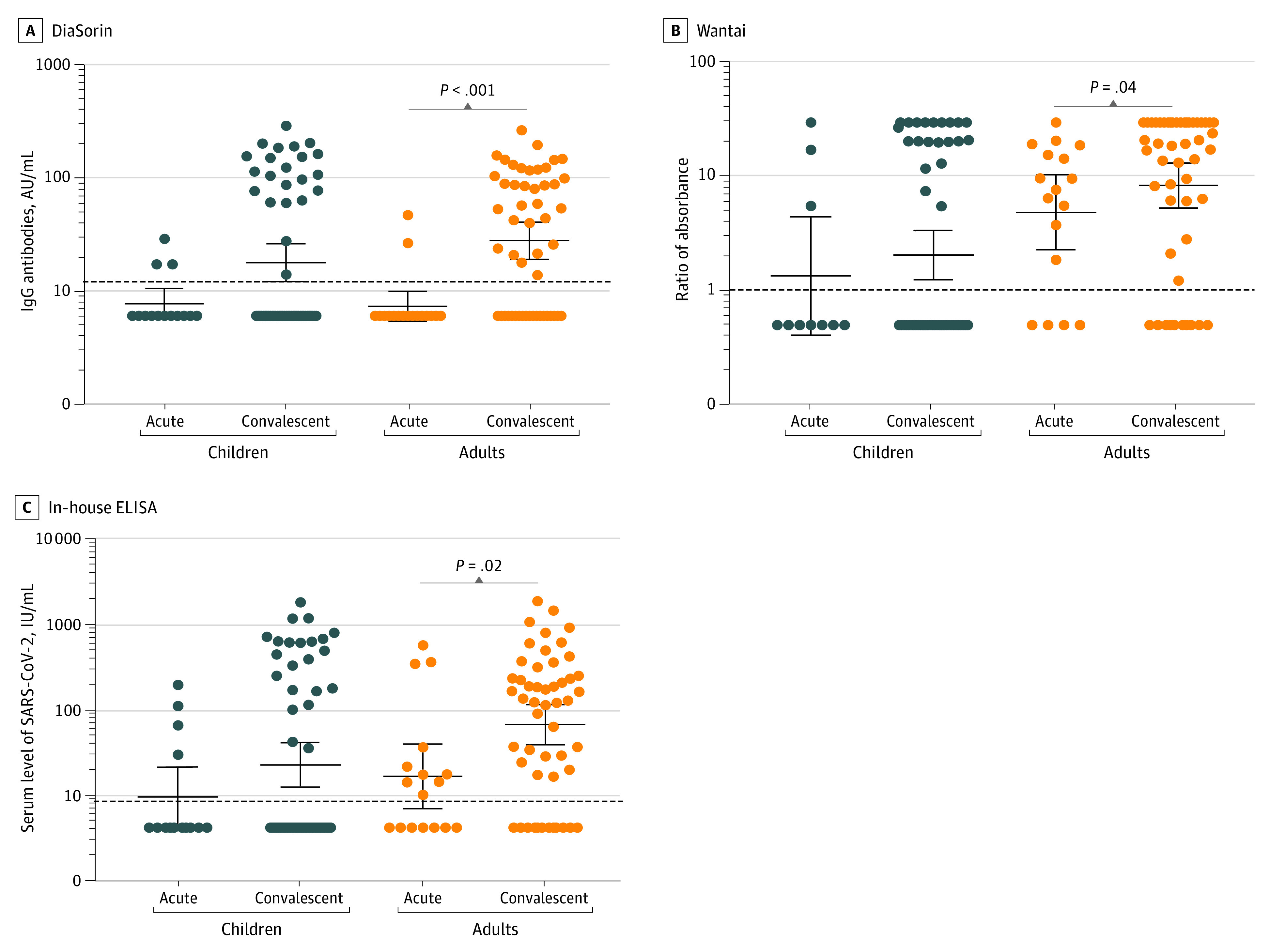
SARS-CoV-2 Antibody Response in Children and Adults Measured by 3 Serological Assays Assays include SARS-CoV-2 S1/S2 immunoglobulin G (DiaSorin), an antibody enzyme-linked immunosorbent assay (ELISA) (Beijing Wantai Biological Pharmacy Enterprise Co, Ltd [Wantai]), and an in-house ELISA. Immunoglobulin G SARS-CoV-2 antibody levels are expressed as means; error bars indicate 95% CIs. Acute levels were measured at a median of 7-12 (IQR, 4-13) days in 14 children and 17 adults. Convalescent levels were measured at a median of 41 (IQR, 31-49) days in 57 children and 51 adults. AU indicates assay units.

**Figure 2.**
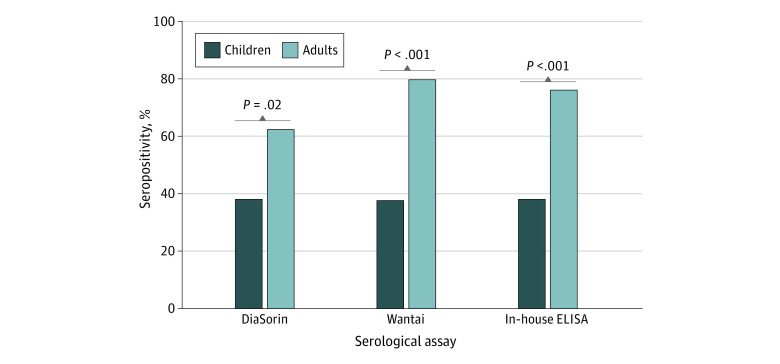
SARS-CoV-2 Immunoglobulin G Seropositivity Rate in Children and Adults at Convalescent Period Measured by 3 Serological Assays Assays include SARS-CoV-2 S1/S2 immunoglobulin G assay (DiaSorin), an antibody enzyme-linked immunosorbent assay (ELISA) (Beijing Wantai Biological Pharmacy Enterprise Co, Ltd [Wantai]), and an in-house ELISA. Convalescent period was a median of 41 (IQR, 31-49) days.

Two commercial assays and 1 in-house ELISA were used to measure antibody responses in children and adults at acute and convalescence periods. There was a significant increase in antibody levels between the acute and convalescence samples for adults but not in children for all 3 assays (eg, in-house ELISA for adults: mean of 84.4 [95% CI, −2.1 to 170.9] IU/mL for acute samples vs 246.1 [95% CI, 139.5-352.6] IU/mL for convalescence samples; *P* = .02). (Figure 1). Results from all 3 assays were highly concordant, with 96 of 108 samples (88.9%) positive by all 3 assays (eFigure 4 in the Supplement) and 94% to 97% agreement between the assays (eTable 3 in the Supplement). A subset of these samples (n = 12) was also tested using a SARS-CoV-2 microneutralization assay, and the results correlated positively with results from all 3 assays (eFigure 5 in the Supplement). Interestingly, lower rates of seropositivity were found in SARS-CoV-2–infected children (22 of 57 [38.6%] for the SARS-CoV-2 S1/S2 IgG assay, 21 of 55 [38.2%] for the SARS-CoV-2 antibody ELISA, and 22 of 57 [38.6%] for the in-house ELISA) at the convalescent time point compared with adults (32 of 51 [62.8%] for the SARS-CoV-2 S1/S2 IgG assay, 40 of 50 [80.0%] for the SARS-CoV-2 antibody ELISA, and 39 of 51 [76.5%] for the in-house ELISA) (Figure 2). No seronegative children at the median convalescent time point (day 41) became seropositive by the median day 94 point (eFigure 6 in the Supplement).

### Factors That May Be Associated With the Antibody Response

**Figure 3.**
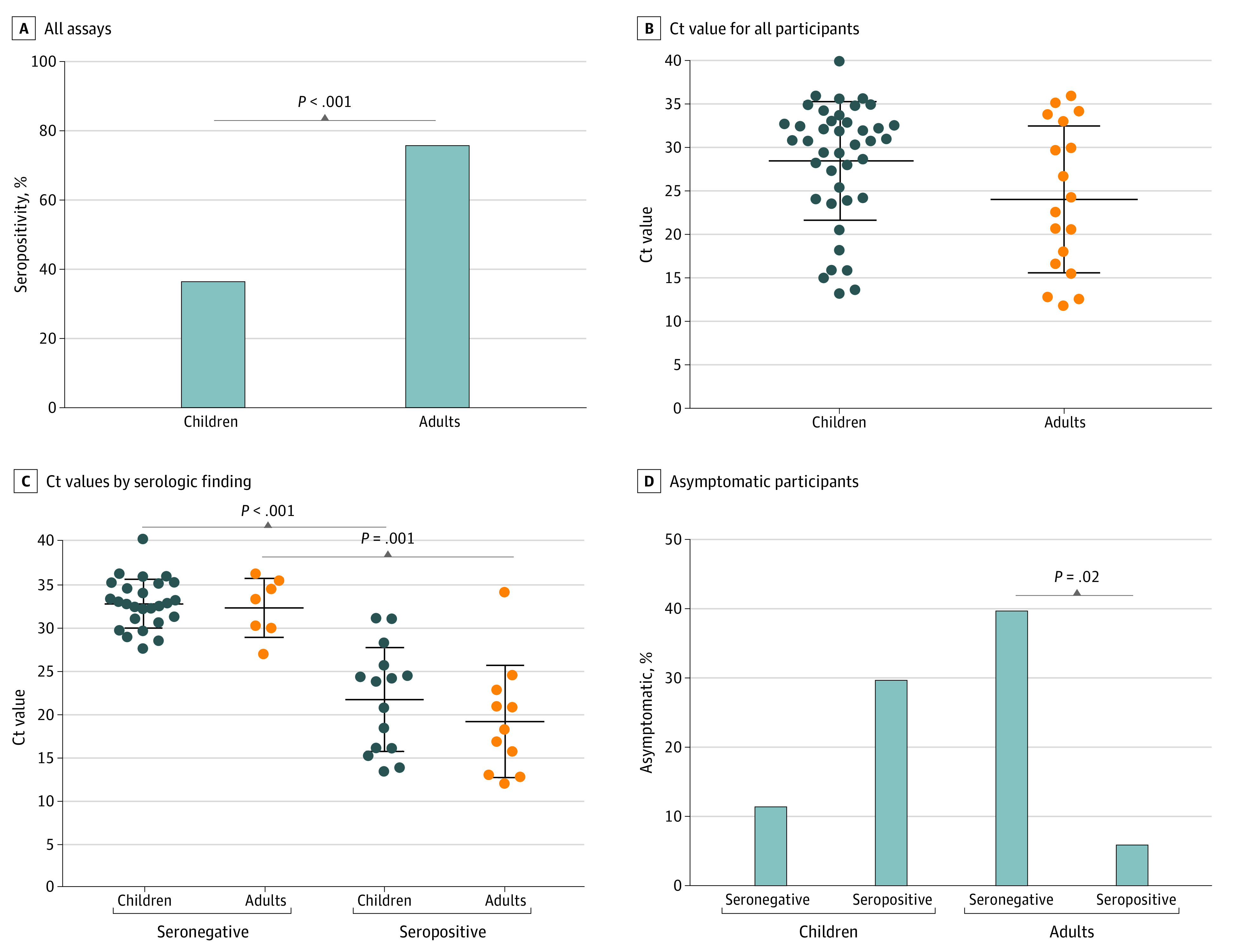
Factors Associated With SARS-CoV-2 Antibody Responses Based on In-House Enzyme-Linked Immunosorbent Assay (ELISA) A, Seropositivity rate in children and adults at convalescent period (median, 41 [IQR, 31-49] days) in 54 children and 42 adults who were seropositive and seronegative by all 3 serological assays. B, Mean (SD) viral load in 42 children and 18 adults with data available. C, Mean (SD) viral load in 42 children and 18 adults stratified by serostatus. D, Proportion of asymptomatic children and adults stratified by serostatus. Ct indicates cycle threshold.

To investigate the factors associated with seroconversion, we included only those patients who were seropositive or seronegative by all 3 serological assays; 9 samples from adults and 3 from children were excluded owing to inconsistent serostatus or lack of testing on all assays owing to sample availability. Based on these criteria, a lower proportion of children had seroconversion to IgG compared with adults (20 of 54 [37.0%] vs 32 of 42 [76.2%]; *P* < .001) (Figure 3A). Although a higher Ct value was found for adults, the difference in viral loads at baseline between children and adults was not statistically significant (mean [SD] Ct value, 28.58 [6.83] vs 24.14 [8.47]; *P* = .09) (Figure 3B). The time from PCR diagnosis to convalescent sampling was also similar between children and adults (median, 41 [IQR, 31-49] vs 41 [IQR, 35-49] days) (eFigure 7 in the Supplement).

Individuals were more likely to be seropositive with higher viral loads and longer viral clearance time (based on those with multiple swab specimens collected), but there were no differences in these parameters between children and adults who were seronegative (mean [SD] Ct value, 32.6 [2.8] in children vs 32.2 [3.4] in adults) or seropositive (mean [SD] Ct value, 21.6 [6.0] in children vs 19.1 [6.5] in adults) (Figure 3C and eFigure 7 in the Supplement). Interestingly, a Ct value of less than 26 was associated with seroconversion in 12 of 15 children (80.0%) and 10 of 11 adults (90.9%). The proportions of children and adults who were seropositive were similar when stratified by sex (children, 10 of 21 girls [47.6%] vs 10 of 33 boys [30.3%]; adults, 18 of 24 women [75.0%] vs 14 of 18 men [77.8%]) (eFigure 7 in the Supplement). A similar age was observed between seronegative and seropositive children (median, 4 [IQR, 2-14] years) as well as between seronegative and seropositive adults (median ages, 37 years for seropositive adults and 40 years for seronegative adults [IQR, 34-39 years]) (eFigure 7 in the Supplement).

When examining the association between symptomatic infection and antibody response, a higher proportion of seronegative adults were asymptomatic compared with seropositive adults (4 of 10 [40.0%] vs 2 of 32 [6.2%]; *P* = .02) (Figure 3D). Symptomatic adults on average had 3 times more antibodies than asymptomatic adults (median, 227.5 [IQR, 133.7-521.6] vs 75.3 [IQR, 36.9-113.6] IU/mL) and higher viral load than asymptomatic adults (mean [SD] Ct value, 24.1 [7.9] vs 32.5 [3.9]), although the number of adults who were asymptomatic and seropositive was small (eFigure 8 in the Supplement). In contrast, a higher proportion of seropositive children were asymptomatic compared with seronegative children (6 of 20 [30.0%] vs 4 of 34 [11.8%]), although this finding was not statistically significant (Figure 3D), and similar levels of antibodies and viral load were observed in children regardless of whether they had any symptoms (eFigure 8 in the Supplement). Notably, viral load correlated with antibody levels in children (*R* = −0.81 [*P* < .001]) and adults (*R* = −0.76 [*P* < .001]), but age did not (*R* = 0.17 [*P* = .21] and *R* = 0.09 [*P* = .59], respectively) (eFigure 8 in the Supplement).

### Cellular Immune Response in Children and Adults After SARS-CoV-2 Infection

**Figure 4.**
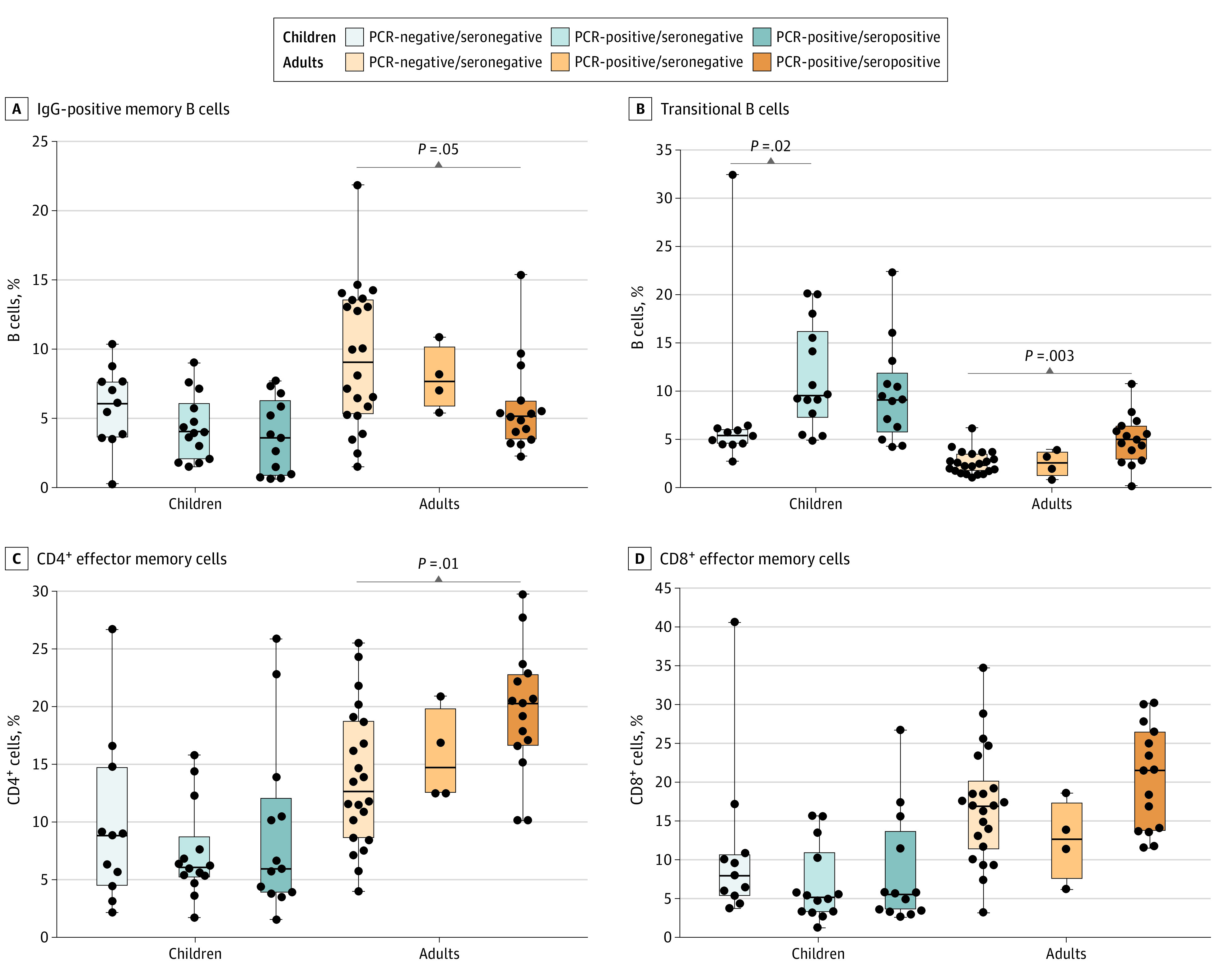
Ex Vivo Cellular Immune Profile During Convalescence Period Among children, 14 had positive polymerase chain (PCR-positive)/seronegative findings; 13, PCR-positive/seropositive findings. Among adults, 4 had PCR-positive/seronegative findings; 15, PCR-positive/seropositive findings. An uninfected control group (PCR-negative/seronegative findings) of 11 children and 22 adults was included for comparison. Bars represent the median; error bars represent the range. IgG indicates immunoglobulin G.

At the convalescent point, compared with uninfected adults, seropositive adults had a significantly lower frequency of IgG-positive memory B cells (median, 5.1% [IQR, 3.5%-6.3%] vs 9.1% [IQR, 9.3%-13.6%]) but higher levels of transitional B cells (5.0% [IQR, 2.9%-6.5%] vs 2.4% [IQR, 1.7%-3.6%]) and CD4^+^ (20.3% [IQR, 16.6%-22.9%] vs 12.7% [IQR, 8.6%-18.8%]) and CD8^+^ T effector memory cells (21.5% [IQR, 13.7%-26.5%] vs 17.0% [IQR, 11.3%-20.3%]). These differences were also observed between seropositive and seronegative adults but were not statistically significant (Figure 4). There were no differences in levels of IgG-positive memory B cells or CD4^+^ or CD8^+^ T effector memory cells in children; however, levels of transitional B cells were higher in seropositive (9.2% [IQR, 5.7%-12.0%) and seronegative (9.5% [IQR, 7.2%-16.2%]) children compared with uninfected children (5.4% [IQR, 4.5%-6.2%]) (Figure 4). No other differences were observed for any of the other T- and B-cell populations examined in children or adults (eFigures 9 and 10 in the Supplement). We also compared innate responses during the acute phase in children and adults. We found no differences in innate immune responses for both children and adults based on serostatus, although the number of samples available for this analysis was small (eFigure 11 in the Supplement).

## Discussion

In this cohort of nonhospitalized patients who were asymptomatic or had mild symptomatic COVID-19, we found that a lower proportion of children with confirmed SARS-CoV-2 infection had seroconversion compared with adults despite no difference in viral load. However, SARS-CoV-2 infection in adults resulted in changes in cellular immune profiles that were most evident in seropositive adults, whereas these changes were not observed in children except for transitional B cells. Taken together, our findings provide insight into how children and adults respond differently to the virus. Our findings also indicate that serological findings may be a less reliable marker of prior SARS-CoV-2 infection, particularly in children. Reduced likelihood of seroconversion may mean that children are less protected against SARS-CoV-2 infections in the long term compared with adults.

Several factors such as age, viral load, sex, comorbidities (including diabetes, cancer, and immunosuppression), and disease severity have been found to be associated with SARS-CoV-2 antibody responses.^29,30,31,32,33,34,35,36,37^ To the best of our knowledge, no data on the proportion of SARS-CoV-2–infected children with seroconversion and the factors impacting this seroconversion have been reported. A recent study^38^ found that 36% of adults with mild COVID-19 did not have seroconversion. In comparison with adults who underwent seroconversion, seronegative adults had a lower viral load in their respiratory tract and were younger (50 vs 40 years).^38^ The proportion of adults without seroconversion was similar to that observed in our study. Other studies in adults^32,39,40,41^ have reported variable seroconversion rates ranging from 5% to 25%. Viral load was similar between children and adults in our cohort, which does not explain why fewer children underwent seroconversion compared with adults. However, our data suggest that a Ct value of less than 26 is associated with seroconversion in both children and adults. A similar Ct value threshold of 25 was found to be associated with seroconversion in a previous study.^42^ Interestingly, asymptomatic infection was associated with lower seropositivity and antibody levels in adults but not in children, consistent with previous studies in adults^37,43^ and children.^9^ This outcome suggests that the host humoral response to SARS-CoV-2 infection in children is different in adults despite similar viral loads and exposure to circulating virus variants.

Several immunological hypotheses as to why children might be less likely to have seroconversion are proposed. First, antibody profiles (antibody isotypes and subclasses)^13,15,44,45,46^ and memory B-cell populations have been reported to be different between children and adults, but this finding has mostly been related to disease severity.^47,48^ We^26^ and others^13,21,44,45,49^ have previously reported similar SARS-CoV-2–specific IgG antibody levels between children and adults. However, we did not measure IgG subclass levels (ie, IgG1 and IgG3), which have also been associated with COVID-19 severity as well as age effects.^15,46^ In our study, we observed a decrease in levels of IgG-positive memory B cells among seropositive adults that corresponded with an increase in transitional B-cell levels. Previous studies have suggested that the activation of preexisting memory B cells during SARS-CoV-2 infection may lead to increased transitional B-cell levels to compensate for the loss in the B-cell compartment.^50,51^ Whether transitional B cells play a role in seroconversion remains to be determined.

Second, T-cell responses differ between SARS-CoV-2–infected children and adults. A recent study^52^ of SARS-CoV-2 T-cell responses in children and adults with mild COVID-19 found that infected children had reduced CD4^+^ T-cell effector memory to SARS-CoV-2 proteins compared with infected adults; this finding is consistent with our results, although we did not undertake ex vivo stimulation experiments. Our data suggest that the difference in seroconversion in children and adults may be due in part to a difference in cellular immune responses (T and B cells).^53,54^ Further studies involving evaluation of SARS-CoV-2–specific cellular responses and/or ex vivo stimulation will be needed to confirm this hypothesis.

Children are thought to have a more robust innate and/or mucosal immune response to SARS-CoV-2 than adults.^14,16,55,56,57^ This response could explain why children in our study did not appear to trigger the adaptive immune system as well as adults. However, our analysis of innate immune responses in children by serostatus did not reveal any differences, likely due to the small sample size. More efficient innate immunity may also suggest a shorter viral clearance time in seronegative children, but this outcome was not observed in our study. A previous study by Tosif et al^55^ showed that the appearance of mucosal SARS-CoV-2 antibody levels in children was associated with symptom resolution and lack of seroconversion in a family case study. Further analysis of mucosal responses in children are ongoing. Clearly, several factors are likely to contribute to the lack of seroconversion, and more studies are needed to improve our understanding of this response.

Our findings have important implications for protection against SARS-CoV-2 in children. Numerous studies have highlighted the importance of antibodies for protection against SARS-CoV-2. A US study of SARS-CoV-2–infected young adults (aged 18-20 years)^58^ reported that SARS-CoV-2–infected seronegative individuals were 80% more likely to be reinfected compared with seropositive individuals. The study also found that low IgG antibody levels in seropositive individuals were associated with reinfection, although seropositive adults had a 10 times lower viral load than reinfected seronegative individuals.^58^ Therefore, a lack of seroconversion may result in a higher susceptibility to reinfection. This hypothesis may have important implications on the transmission of SARS-CoV-2 in the community and the public health response.

It is important to note that our findings are based on the ancestral Wuhan SARS-CoV-2 virus that was circulating at the time of our study in 2020. Whether a lower seroconversion rate in children is also observed after infection with the SARS-CoV-2 Delta or Omicron variants is unknown and warrants further research. The Delta variant has been associated with a 1000-fold higher viral load compared with the Wuhan strain,^59^ so a higher seroconversion rate in children might be expected, while studies have shown that Omicron is even more infectious than the Delta variant.^6^

### Limitations

Limitations of this study include the small sample size, particularly for the cellular analyses. In addition, the results from our cohort of children and adults with mild COVID-19 may not be generalizable to other study populations, such as older adults or individuals with underlying medical conditions. Other limitations include the antibody subclasses such as IgA, antibodies against other SARS-CoV-2 proteins other than the spike protein (ie, N protein), and SARS-CoV-2–specific cellular immune responses that were not measured. Nevertheless, for the serological responses, the 3 assays used in this study are established assays for measuring SARS-CoV-2 serology and IgG, which is known to strongly correlate with neutralizing antibodies.^26,60,61^

## Conclusions

To our knowledge, this cohort study is the first to document a lower proportion of children with seroconversion compared with adults despite a similar clinical and virological profile. Seronegative children have a greater potential risk of reinfection. Our findings have important implications for public health responses in controlling SARS-CoV-2 infection among children and support COVID-19 vaccination strategies once priority groups have been vaccinated.

## Notes

Supplement. **eTable 1.** Antibody Cocktail to Identify Adaptive Immune Cell Populations **eTable 2.** Antibody Cocktail to Identify Innate Immune Cell Populations **eTable 3.** Concordance of 3 Serological Assays for All Samples at Convalescent Period From the Whole Household Cohort Study **eFigure 1.** Gating Strategy to Identify T-cell Subsets **eFigure 2.** Gating Strategy to Identify B-cell/TFH Subsets **eFigure 3.** Gating Strategy for Innate Cell Populations **eFigure 4.** Correlation Analysis of 3 Serological Assays **eFigure 5.** Correlation Analysis of 3 Serological Assays Against SARS-CoV-2 Microneutralization Assay **eFigure 6.** SARS-CoV-2 IgG Levels Over Time in Children and Adults Using an In-house ELISA **eFigure 7.** Duration of Post-PCR Diagnosis and Viral Clearance, Sex, and Age Associated With SARS-CoV-2 Antibody Responses **eFigure 8.** Symptoms and Correlation Analysis Associated With SARS-CoV-2 Antibody Responses **eFigure 9.** Humoral Immune Cells Profile During Convalescence Period in Children and Adults Following SARS-CoV-2 Infection **eFigure 10.** Cellular Immune Profile (T Cells) During Convalescence Period in Children and Adults Following SARS-CoV-2 Infection **eFigure 11.** Innate Cell Profiles During Acute Phase for Children and Adults Following SARS-CoV-2 Infection
