# Supplementary material for: Comparison of Seroconversion in Children and Adults With Mild COVID-19
Source: JAMA Netw Open. 2022 Mar 9;5(3):e221313. doi: 10.1001/jamanetworkopen.2022.1313 (PMC8908077; doi:10.1001/jamanetworkopen.2022.1313)
Supplement: Supplement. — eTable 1. Antibody Cocktail to Identify Adaptive Immune Cell Populations eTable 2. Antibody Cocktail to Identify Innate Immune Cell Populations eTable 3. Concordance of 3 Serological Assays for All Samples at Convalescent Period From the Whole Household Cohort Study eFigure 1. Gating Strategy to Identify T-cell Subsets eFigure 2. Gating Strategy to Identify B-cell/TFH Subsets eFigure 3. Gating Strategy for Innate Cell Populations eFigure 4. Correlation Analysis of 3 Serological Assays eFigure 5. Correlation Analysis of 3 Serological Assays Against SARS-CoV-2 Microneutralization Assay eFigure 6. SARS-CoV-2 IgG Levels Over Time in Children and Adults Using an In-house ELISA eFigure 7. Duration of Post-PCR Diagnosis and Viral Clearance, Sex, and Age Associated With SARS-CoV-2 Antibody Responses eFigure 8. Symptoms and Correlation Analysis Associated With SARS-CoV-2 Antibody Responses eFigure 9. Humoral Immune Cells Profile During Convalescence Period in Children and Adults Following SARS-CoV-2 Infection eFigure 10. Cellular Immune Profile (T Cells) During Convalescence Period in Children and Adults Following SARS-CoV-2 Infection eFigure 11. Innate Cell Profiles During Acute Phase for Children and Adults Following SARS-CoV-2 Infection [file jamanetwopen-e221313-s001.pdf]

Supplementary Online Content

Toh ZQ, Anderson J, Mazarakis N, et al. Comparison of seroconversion in children and adults with mild COVID-19. *JAMA Netw Open*. 2022;5(3):e221313. doi:10.1001/jamanetworkopen.2022.1313

eTable 1. Antibody Cocktail to Identify Adaptive Immune Cell Populations
eTable 2. Antibody Cocktail to Identify Innate Immune Cell Populations
eTable 3. Concordance of 3 Serological Assays for All Samples at Convalescent Period From the Whole Household Cohort Study
eFigure 1. Gating Strategy to Identify T-cell Subsets
eFigure 2. Gating Strategy to Identify B-cell/TFH Subsets
eFigure 3. Gating Strategy for Innate Cell Populations
eFigure 4. Correlation Analysis of 3 Serological Assays
eFigure 5. Correlation Analysis of 3 Serological Assays Against SARS-CoV-2 Microneutralization Assay
eFigure 6. SARS-CoV-2 IgG Levels Over Time in Children and Adults Using an In-house ELISA
eFigure 7. Duration of Post-PCR Diagnosis and Viral Clearance, Sex, and Age Associated With SARS-CoV-2 Antibody Responses
eFigure 8. Symptoms and Correlation Analysis Associated With SARS-CoV-2 Antibody Responses
eFigure 9. Humoral Immune Cells Profile During Convalescence Period in Children and Adults Following SARS-CoV-2 Infection
eFigure 10. Cellular Immune Profile (T Cells) During Convalescence Period in Children and Adults Following SARS-CoV-2 Infection
eFigure 11. Innate Cell Profiles During Acute Phase for Children and Adults Following SARS-CoV-2 Infection

This supplementary material has been provided by the authors to give readers additional information about their work.

© 2022 Toh ZQ et al. *JAMA Network Open*.

eTable 1. Antibody Cocktail to Identify Adaptive Immune Cell Populations

Antibody Cocktail 1	Supplier	Antibody Cocktail 2	Supplier
CD3-BUV395	BD Bioscience, San Diego, CA, USA	CD3-Percp/Cy5.5	BD Bioscience, San Diego, CA, USA
CD4-BV421	BD Bioscience, San Diego, CA, USA	CD4-BV510	BioLegend, San Diego, USA
CD8-BUV805	BD Bioscience, San Diego, CA, USA	CXCR5-APCR700	BD Bioscience, San Diego, CA, USA
CD45RA-Percp/Cy5.5	BD Bioscience, San Diego, CA, USA	PD-1-PEcy7	BD Bioscience, San Diego, CA, USA
CCR7-BV785	BioLegend, San Diego, USA	CD19-BV785	BioLegend, San Diego, USA
CD69-BV650	BD Bioscience, San Diego, CA, USA	CD20-BV421	BioLegend, San Diego, USA
HLA-APC-H7	BD Bioscience, San Diego, CA, USA	CD27-BUV737	BD Bioscience, San Diego, CA, USA
Zombie NIR	BioLegend, San Diego, USA	CD19-BV785	BioLegend, San Diego, USA
		CD38-BUV496	BD Bioscience, San Diego, CA, USA
		CD24-BV711	BioLegend, San Diego, USA
		IgG-BV605	BD Bioscience, San Diego, CA, USA
		IgM-FITC	BioLegend, San Diego, USA
		Zombie NIR	BioLegend, San Diego, USA

© 2022 Toh ZQ et al. *JAMA Network Open*.

eTable 2. Antibody Cocktail to Identify Innate Immune Cell Populations

Surface Marker	Fluorophore	Clone	Final Dilution
CD14	BV786	M5E2	1:50
CD45	BV711	HI30	1:100
CD56	BUV737	NCAM16.2	1:100
CD11c	PE-Cy7	B-ly6	1:100
CD3	BB515	UCHTI	1:100
CD15	PE-CF594	W6D3	1:200
HLA-DR	V500	G46-6	1:200
CD19	BV605	SJ25C1	1:200
CD16	BUV395	3G8	1:400
Live/dead	N-IR		

© 2022 Toh ZQ et al. *JAMA Network Open*.

eTable 3. Concordance of 3 Serological Assays for All Samples at Convalescent Period From the Whole Household Cohort Study

		**Diasorin**					**Diasorin**					**Wantai**	
		Positive	Negative				Positive	Negative				Positive	Negative
**In-house ELISA**	Positive	56	10		**Wantai**	Positive	54	10		**In-house ELISA**	Positive	61	3
	Negative	3	164			Negative	3	162			Negative	3	162
	Total		233			Total		229			Total		229
Agreement		0.94					0.94					0.97	

© 2022 Toh ZQ et al. *JAMA Network Open*.

eFigure 1. Gating Strategy to Identify T-cell Subsets From live single cells, T-cells were identified by positive CD3 expression. CD4+ and CD8+ T-cells were identified from the CD3+ population. CD4+ T-cells and CD8+ T-cells were characterised into naïve (N), effector (E), central memory (CM) and effector memory (EM) based off CCR7 and CD45RA expression. These were CCR7+CD45RA+, CCR7-CD45RA+, CCR7+CD45RA- and CCR7-CD45RA- respectively. CD69+ and HLA+ expression was also characterised on CD4+ and CD8+ T-cells.

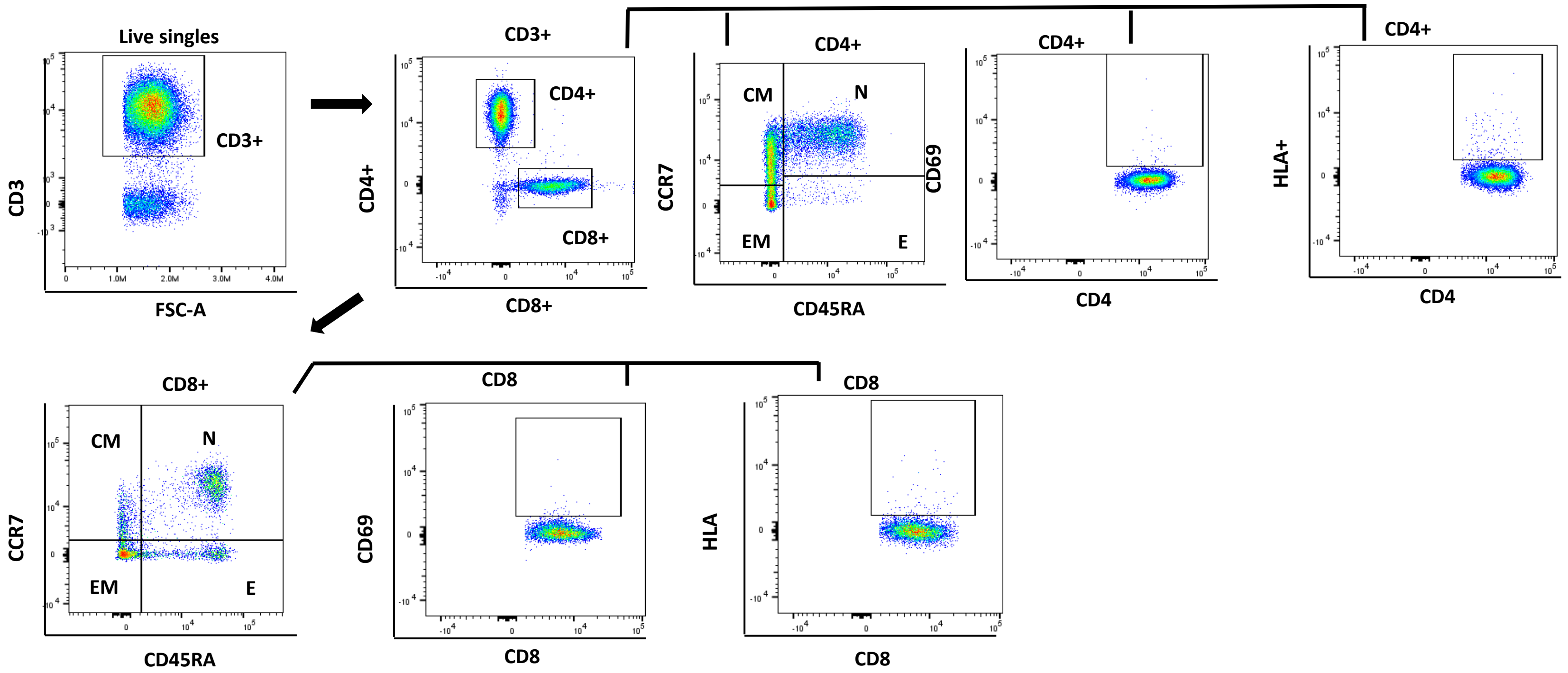

© 2022 Toh ZQ et al. *JAMA Network Open*.

eFigure 2. Gating Strategy to Identify B-cell/T_{FH} Subsets From live single cells, T-cells were identified as CD3+CD19- and B-cells were identified as the CD3-CD19+ population. From the CD3+ population, T_{FH} was characterised as CD4+CXCR5+PD-1+ expressing T-cells. From the B-cell population, memory was identified as CD27+, transitional B-cells as CD24+CD38+ and plasmablasts as CD27+CD20-CD38+. IgM and IgG expression were also characterised on B-cell populations.

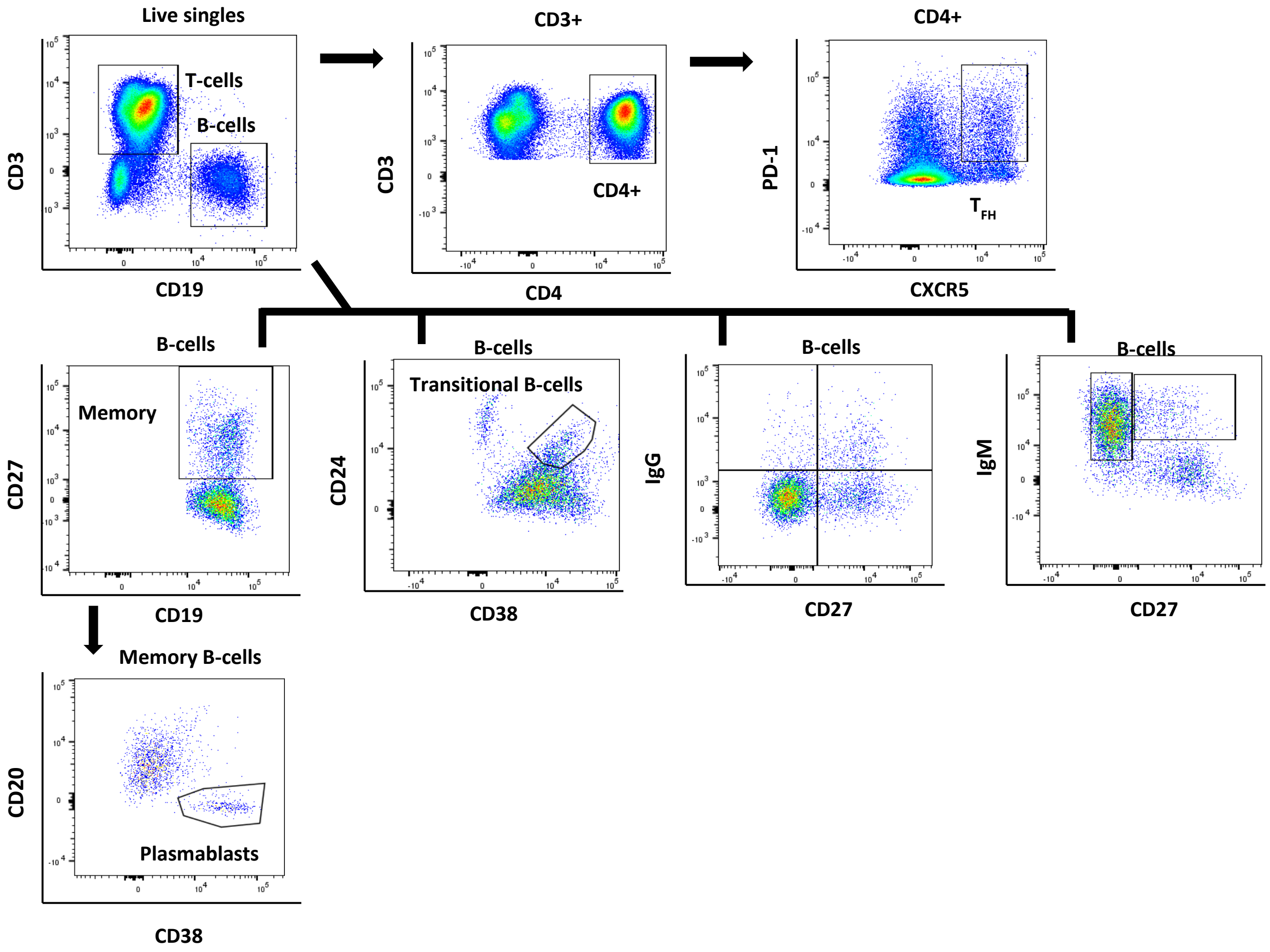

© 2022 Toh ZQ et al. *JAMA Network Open*.

eFigure 3. Gating Strategy for Innate Cell Populations Granulocytes were selected within CD45+ leukocytes based on their SSC profile and CD15 expression. Neutrophils were CD15+CD16+ and eosinophils were CD15+CD16-. Within the non-granulocyte fraction, CD3 T cells were identified and B cells were identified based on CD19 and HLA-DR expression. CD11c+CD14+ monocytes were gated within the non-T cell and non-B cell fraction. Total dendritic cells were HLADR+CD11c+CD14- and NK cells were HLADR-CD3-CD56+ cells.

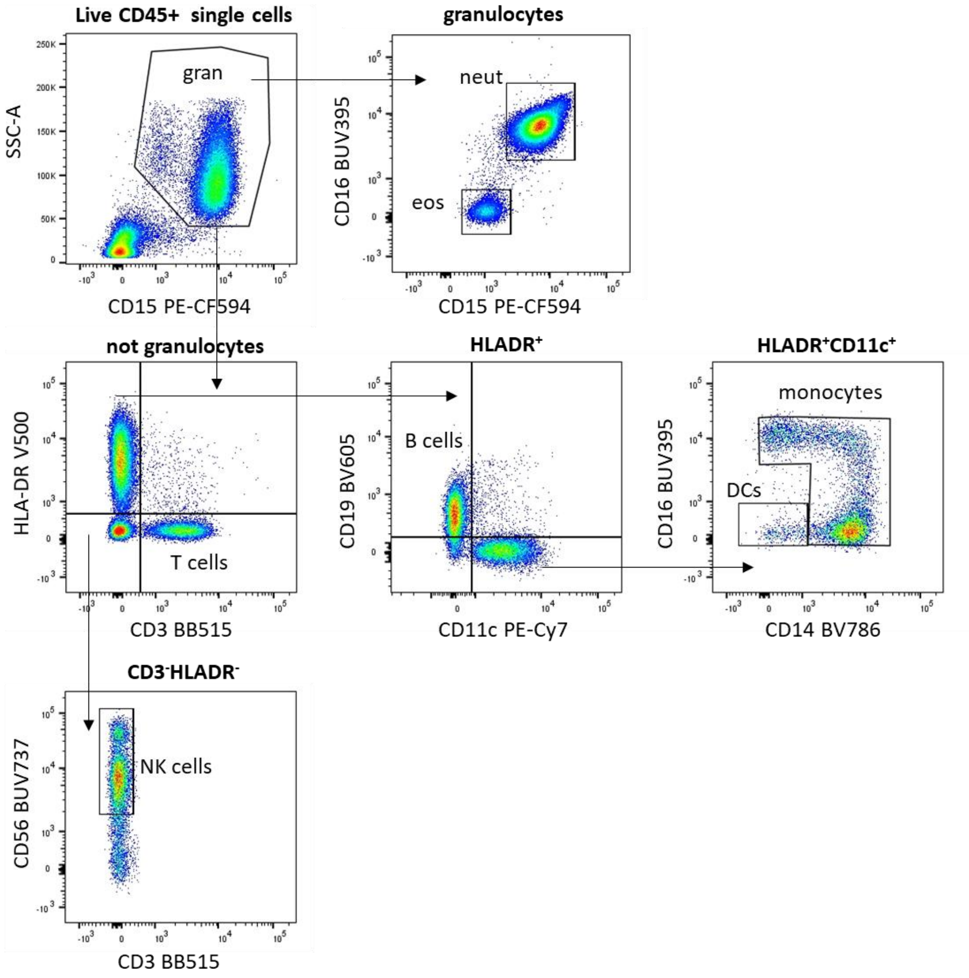

© 2022 Toh ZQ et al. *JAMA Network Open*.

eFigure 4. Correlation Analysis of 3 Serological Assays

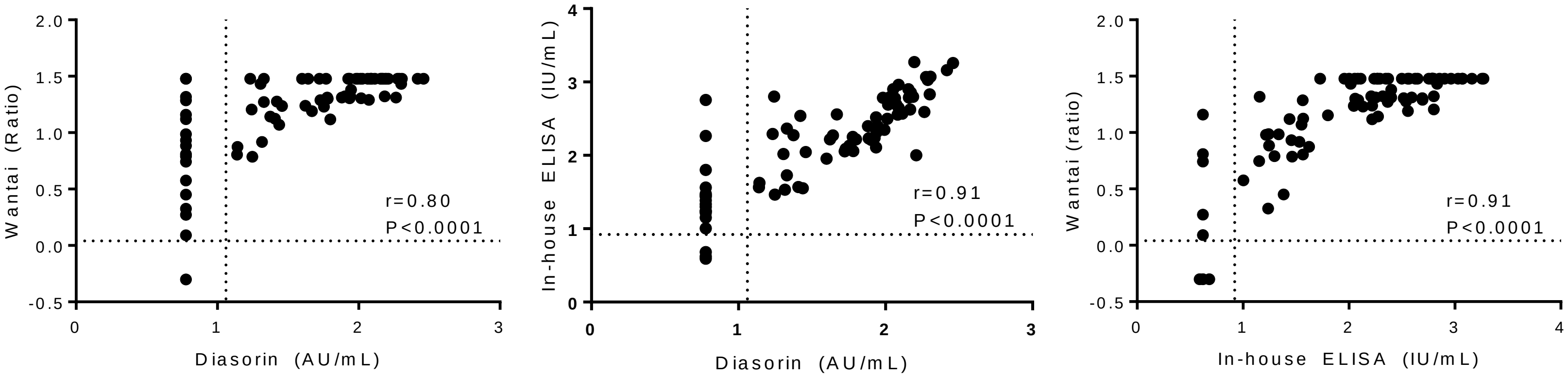

Antibody levels were log-transformed and analysed using Pearson correlation analyses. N=138-145.

© 2022 Toh ZQ et al. *JAMA Network Open*.

eFigure 5. Correlation Analysis of 3 Serological Assays Against SARS-CoV-2 Microneutralization Assay

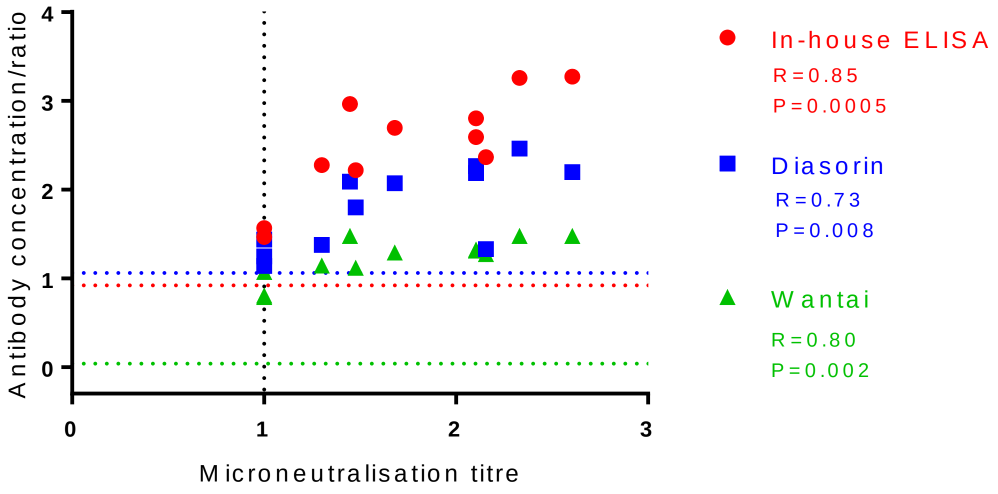

Antibody levels were log-transformed and analysed using Pearson correlation analyses. Coloured dotted line represents assay specific cut-off for seropositivity. N=12.

© 2022 Toh ZQ et al. *JAMA Network Open*.

eFigure 6. SARS-CoV-2 IgG Levels Over Time in Children and Adults Using an In-house ELISA

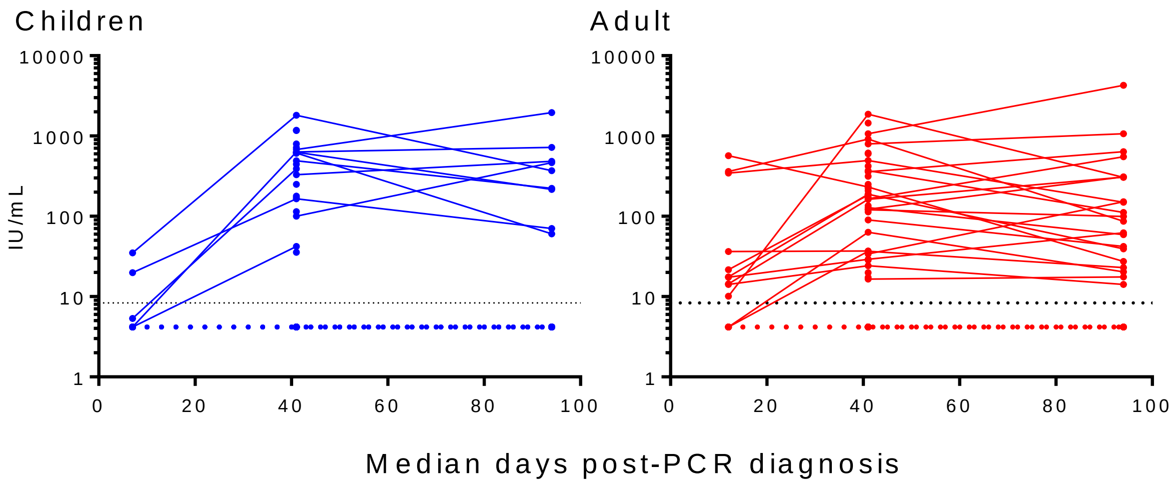

Number of samples per timepoint: Children, Day 7 (N=13), Day 41 (N=59), Day 94 (N=26); Adult, Day 12 (N=20), Day 41 (N=57), Day 94 (N=29).

© 2022 Toh ZQ et al. *JAMA Network Open*.

eFigure 7. Duration of Post-PCR Diagnosis and Viral Clearance, Sex, and Age Associated With SARS-CoV-2 Antibody Responses

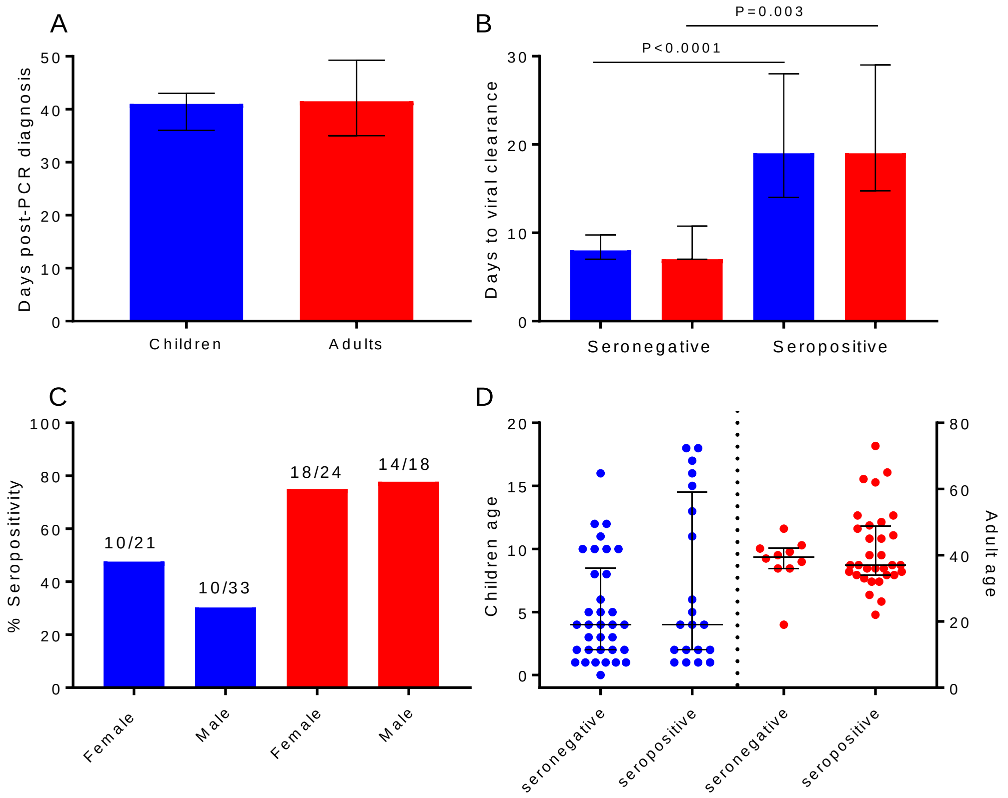

(A) Median days (IQR) between positive PCR diagnosis and convalescent blood sampling between children (N=54) and adult (N=42). (B) Duration of viral clearance (median days, IQR) stratified by serostatus (seronegative children, N=20, seropositive children, N=7; seronegative adult, N=4, seropositive adult, N=10). (C) Seropositivity rate stratified by sex. (D) Age of children and adults stratified by serostatus (Children, N=54; Adult, N=42) (Median, IQR).

© 2022 Toh ZQ et al. *JAMA Network Open*.

eFigure 8. Symptoms and Correlation Analysis Associated With SARS-CoV-2 Antibody Responses

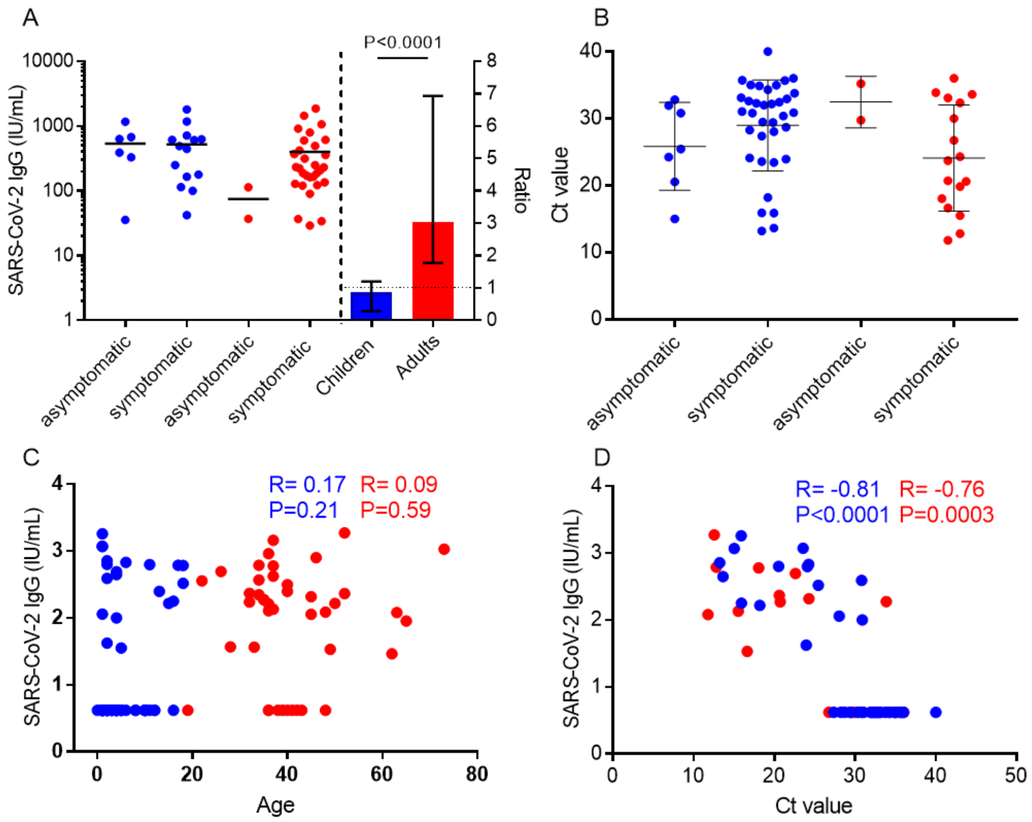

(A) Median antibody levels (IQR) based on symptoms (left y-axis) and median fold-change in antibody levels between asymptomatic and symptomatic in children (N=6 vs N=14) and adults (N=2 vs N=30) (right y-axis). (B) Mean viral load (SD) stratified based on symptoms in children (asymptomatic, N=7 vs symptomatic, N=35) and adults (asymptomatic, N=2 vs symptomatic, N=17). (C) Correlation between antibody levels and viral load. (D) Correlation between antibody levels and age. Blue dots/bars represent children and red dots/bars represent adults. Seropositivity was defined as seropositive by all three assays. Pearson's correlation analysis was used to examine association. Ct value: cycle threshold.

© 2022 Toh ZQ et al. *JAMA Network Open*.

eFigure 9. Humoral Immune Cells Profile During Convalescence Period in Children and Adults Following SARS-CoV-2 Infection

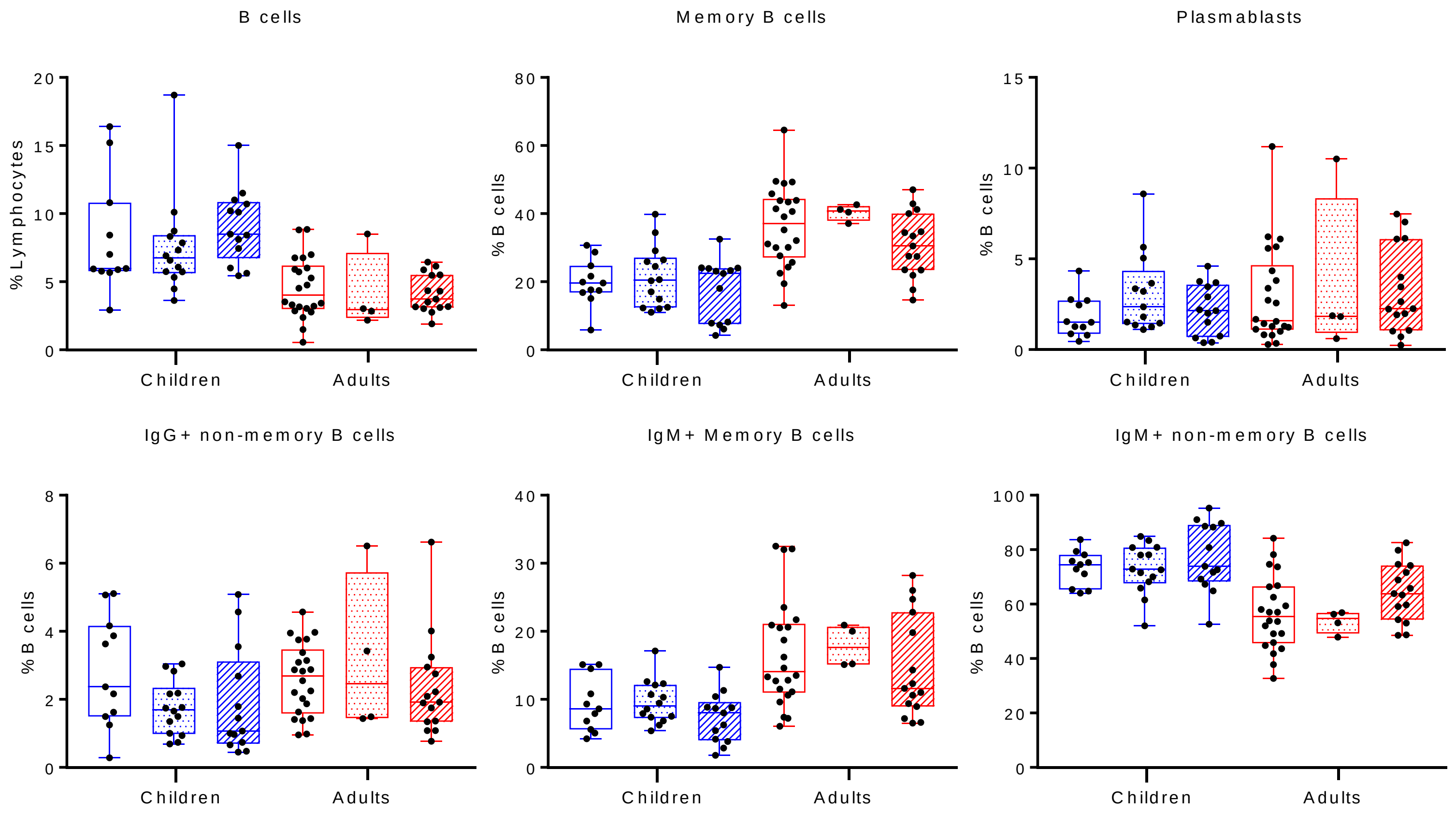

Convalescence period (median day 41) in children (PCR+sero-, N=14; PCR+sero+, N=13) and adults (PCR+sero-, N=4, PCR+sero+, N=15) following SARS-CoV-2 infection. An uninfected control group was included for comparison (PCR-sero-: children, N=11; adults, N=22). Bars represent median and range.

© 2022 Toh ZQ et al. *JAMA Network Open*.

eFigure 10. Cellular Immune Profile (T Cells) During Convalescence Period in Children and Adults Following SARS-CoV-2 Infection

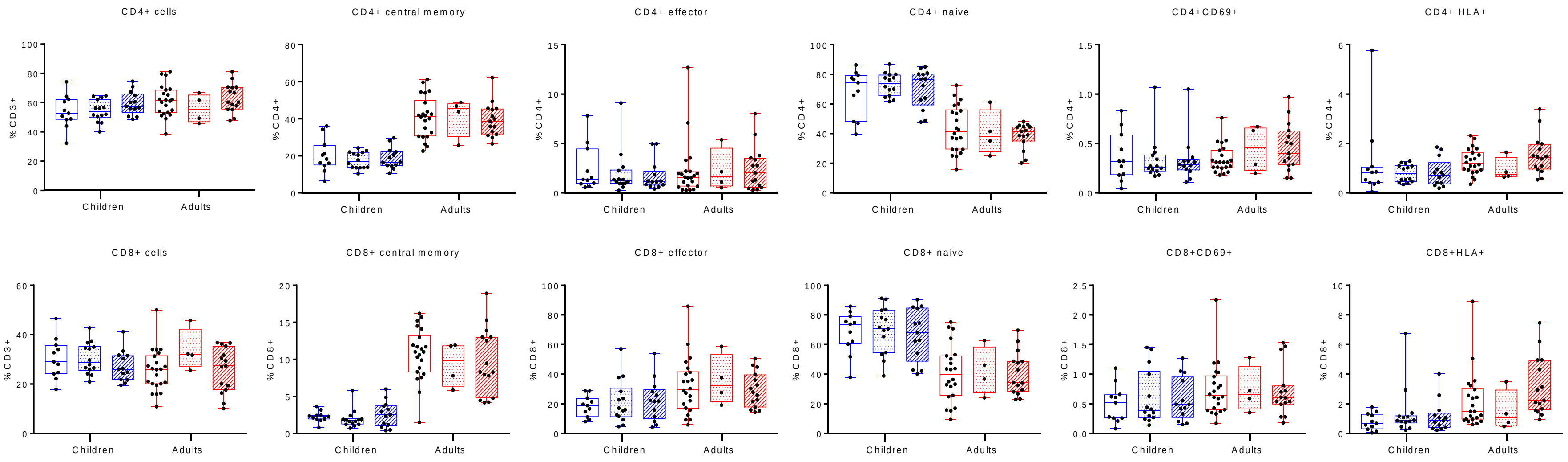

Convalescence period (median day 41) in children (PCR+sero-, N=14; PCR+sero+, N=13) and adults (PCR+sero-, N=4, PCR+sero+, N=15) following SARS-CoV-2 infection. An uninfected control group was included for comparison (PCR-sero-: children, N=11; adults, N=22). Bars represent median and range.

© 2022 Toh ZQ et al. *JAMA Network Open*.

eFigure 11. Innate Cell Profiles During Acute Phase for Children and Adults Following SARS-CoV-2 Infection

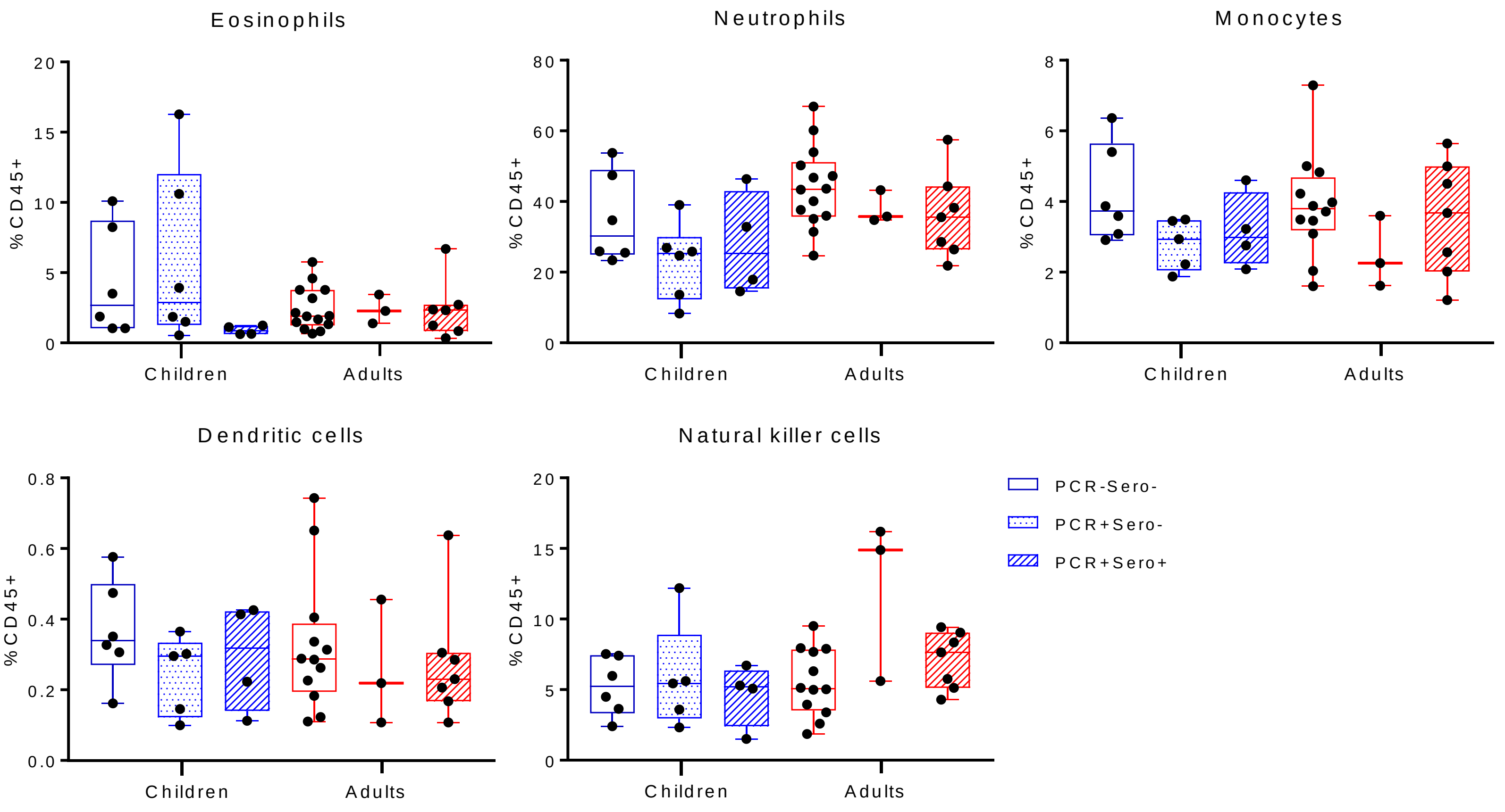

© 2022 Toh ZQ et al. *JAMA Network Open*.

Acute phase (day 7-12) for children (PCR+sero-, N=7; PCR+sero+, N=4) and adults (PCR+sero-, N=3, PCR+sero+, N=8) following SARS-CoV-2 infection. An uninfected control group was included for comparison (PCR-sero-: children, N=6; adults, N=16). Bars represent median and range.

© 2022 Toh ZQ et al. *JAMA Network Open*.
